# Key Insights into Respiratory Virus Testing: Sensitivity and Clinical Implications

**DOI:** 10.3390/microorganisms13010063

**Published:** 2025-01-02

**Authors:** Julio Garcia-Rodriguez, Frédéric Janvier, Clemens Kill

**Affiliations:** 1 Unidad de Enfermedades Infecciosas, Hospital Universitario La Paz, 28046 Madrid, Spain; jgarciarodriguez@salud.madrid.org; 2 Service de Microbiologie et Hygiène Hospitalière, Hôpital d’Instruction des Armées Sainte Anne, 83000 Toulon, France; 3 Zentrum für Notfallmedizin, Universitätsmedizin Essen, 45147 Essen, Germany; clemens.kill@uk-essen.de

**Keywords:** SARS-CoV-2, influenza A, influenza B, respiratory syncytial virus, polymerase chain reaction, cycle threshold, viral load, rapid antigen tests

## Abstract

Acute respiratory infections are a significant challenge in primary care and hospital settings. Viruses are the most common etiology and the overlapping symptomatology among major respiratory viruses, such as influenza, severe acute respiratory syndrome coronavirus 2, and respiratory syncytial virus, requires the use of diagnostic tests that deliver early and accurate results. With the increasing availability of rapid antigen tests (RATS), it is tempting to prefer them over polymerase chain reaction (PCR) tests. However, compelling arguments support the existing recommendations in some European countries to maintain PCR testing for patient management throughout the year. RATs show sensitivities below 30% with lower viral loads, which are common and can have significant clinical implications. RATs perform well at lower cycle threshold (Ct) values, with sensitivity reaching 97.9% for Ct values below 20, which drops significantly for values above 25. Factors affecting viral load include disease stage, vaccination status, and viral variants, all of which can compromise the accuracy of antigen tests. Multi-target PCR tests effectively overcome these issues, ensuring reliable diagnosis. Additionally, the early detection of paucisymptomatic cases is essential in primary care and hospital settings to facilitate isolation and prevent secondary infections. Economic analyses support the use of comprehensive PCR tests, such as triplex-type tests, detecting SARS-CoV-2, influenza viruses, and RSV, as a first-line approach, as they can reduce case numbers and healthcare resource utilization. Maintaining PCR testing year-round is therefore crucial for the effective management of respiratory infections.

## 1. Introduction

Acute respiratory infectious diseases pose a severe threat to public health, impacting morbidity and mortality rates while exerting pressures on healthcare systems, from primary care clinicians to hospital networks. Influenza viruses, human coronaviruses, respiratory syncytial virus, rhinoviruses, metapneumoviruses, and parainfluenza viruses are the main viruses responsible for these respiratory infections. However, considering the available specific antiviral drugs, prevalence, and reportability [1], severe acute respiratory syndrome coronavirus 2 (SARS-CoV-2), influenza A and B, and respiratory syncytial virus (RSV) are viruses of concern and form the focus of this article [2]. According to the 2024 VACCELERATE consortium survey, 78.6% of infectious disease experts rank influenza as the top pandemic risk, while SARS-CoV-2 also ranked high due to its history of pandemic outbreak, underscoring their likelihood of causing the next pandemic [3].

## 2. Current Situation—Incidence and Outcome

Global influenza activity surged in late 2021 and throughout 2022, rebounding from a significant dip in 2020. This resurgence manifested in atypical patterns, with delayed flu seasons in the Northern Hemisphere and unexpectedly early outbreaks in the Southern Hemisphere [4], resulting in out-of-season consultations that could be attributable to resumed global travel and reduced pandemic-era public health measures. Indeed, the 2022/2023 influenza season in the European Union/European Economic Area (EU/EEA) marked a return to pre-pandemic patterns, characterized by an earlier start and peak, predominantly driven by the A(H3N2) strain, along with A(H1N1)pdm09, the influenza virus responsible for the 2009 pandemic and type B viruses. Particularly alarming is the absence of the influenza B/Yamagata lineage since April 2020 and the reduced genetic diversity in key flu viruses such as A(H3N2), A(H1N1), and B/Victoria. This scenario suggests potential challenges ahead: reduced population immunity and virus mutations may lead to more severe flu seasons and reduced vaccine effectiveness [1,5]. Correspondingly, in France, the climax of bronchiolitis and RSV cases in late November 2023 returned to levels seen before the pandemic, with 44.3% of hospitalizations involving children under two years [6]. In Denmark, the relaxation of coronavirus disease 2019 (COVID-19) precautions in spring 2021 triggered a significant atypical outbreak of RSV [7].

Meanwhile, the ongoing emergence of COVID-19 Omicron subvariants and a decrease in testing frequency continue to challenge public health in Europe. The continent faces considerable excess mortality, with 467,921 deaths reported in 2022 [8]. Additionally, long COVID-19 remains a critical issue, affecting between 3% and 20% of those infected, with prolonged work absences continuing to impact economic output [8].

For clinicians, decisively diagnosing whether a patient is afflicted by a specific viral infection carries significant consequences not only for the individual’s treatment plan but also for public health. This article highlights the importance of accurate testing, emphasizing how the timing of symptom onset, the quality of the sample, and the patient’s immune status significantly influence test accuracy. The authors will focus on patients presenting in primary care, emergency departments, and medical wards. Intensive care unit patients are out of the scope of this present article. The approach ensures that if a patient tests accurately for a virus, early specific treatment and infection prevention measures can effectively manage the illness, reducing transmission risk, particularly in paucisymptomatic cases who can transmit the infection onwards, creating nosocomial clusters from the emergency department to wards [6].

## 3. Diagnosis and Treatment—Early and Accurate Diagnosis Might Be Challenging but Enables Timely and Efficient Management of Cases

When faced with patients showing symptoms of acute respiratory infection, identifying the responsible virus is a challenge [9,10]. Given the similarity in symptoms across these infections, distinguishing between RSV, influenza A and B, and SARS-CoV-2 requires specific diagnostic tests [11,12,13]. As most of the currently available diagnostic tools are performed on nasopharyngeal samples, the authors will focus on upper respiratory tract collection sites. Hence, to be precise, the data presented here were obtained from these sites.

Indeed, accurate and early diagnostic testing allows for improved patient outcomes and helps to prevent secondary cases; fast testing is useful not just to inform clinical decisions but also to start infection prevention and control (IPC) measures. This approach prioritizes symptomatic individuals, those who have been in contact with confirmed cases, areas experiencing severe outbreaks, and even asymptomatic individuals. As these viruses are circulating at the same time, patients presenting with acute respiratory symptoms should be tested for at least SARS-CoV-2 and influenza, and even RSV in children and older patients [14]. Once a positive result is confirmed, isolation and IPC protocols can be adapted to prevent nosocomial clusters in hospitals and secondary cases in community settings [6,15]. The early initiation of specific antiviral therapy has been shown to improve patient outcomes, including mortality. Both the Centers for Disease Control and Prevention and the European Center for Disease Prevention and Control (ECDC) recommend prompt antiviral treatment. Oseltamivir must be started within 48 h of illness onset for patients confirmed to have influenza, or those suspected of having it and who are at a higher risk of complications [16,17]. Nirmatrelvir/ritonavir should be administered as early as possible post-COVID-19 diagnosis and within 5 days from the onset of symptoms to decrease the risk of severe outcomes, including hospitalization and death [18,19].

Management of paucisymptomatic individuals represents an important challenge, especially from a patient-centered and IPC standpoint. True symptomatic presentations for SARS-CoV-2 occur mainly around the height of viral replication, marking this phase where patients are most frequently assessed and tested. This period is critical as the presence of viral nucleic acids is most readily detectable by standard diagnostic testing, whereas in early testing, even the most sensitive test is required to detect SARS-CoV-2 due to very low viral nucleic acid concentrations. As the infection progresses beyond the symptomatic peak, the levels of replication-competent virus diminish rapidly. From nasopharyngeal swabs, the window during which a pathogen is detectable typically spans approximately 5 to 7 days post-symptom onset. Beyond this period, the concentration of actively replicating virus significantly decreases and is often undetectable, coinciding generally with the resolution of symptoms in an otherwise healthy individual [20,21,22]. Additionally, as indicated by the cycle threshold (Ct), initial tests of SARS-CoV-2 patients often reveal that the majority are past the peak of viral shedding in samples collected from the upper respiratory tract [20]. By definition, loosely inversely proportional to the viral load in the specimen, a lower Ct value indicates a higher concentration of the virus, with Ct values below 40 generally considered positive in PCR tests [23,24]. Specimens from vaccinated individuals with breakthrough acute respiratory infections often display higher Ct values, which may reduce the detection efficiencies and affect sensitivities [25,26]. While RSV vaccination is new, preliminary data show that it can significantly reduce the amount of virus, especially in infants and older adults [27]. Although detailed data for influenza are less clear, vaccines are known to lessen illness severity and duration, likely leading to lower viral loads in vaccinated individuals [28].

## 4. SARS-CoV-2 Infection Control—Complexity of Transmission

From an IPC perspective, asymptomatic patients may have the lowest viral loads but can shed the virus for up to 8 days, creating a transmission risk [29]. Studies show that the likelihood of flu transmission within a household setting can increase to approximately 38%, with asymptomatic individuals transmitting the infection to as many as 6% or 25% of household contacts. This intrahousehold spread implicates children as vectors within the family unit, contributing to approximately 27% of secondary infections stemming from exposure to an infected child [30]. These emphasize the non-direct correlation between viral load and symptom severity or recovery, stressing the challenge that when asymptomatic or paucisymptomatic patients transmit infections, it could bypass contact tracing efforts aimed at controlling transmission [30,31,32,33]. For RSV, studies show that 42% of RSV cases were asymptomatic when tested regularly, and 18% of close contacts to a hospitalized RSV were asymptomatically infected. Further, approximately 30% of the observed RSV secondary cases acquired the infection prior to the onset of symptoms in the index case, typically before the fourth day when the RSV viral load is at its lowest, suggesting that low viral load can transmit infections since the viral load peaks on approximately day 6 [34]. A recent study in an emergency department focusing on routine testing for SARS-CoV-2, Flu A and B, and RSV showed a significant number of RSV infections in adults with symptoms of respiratory infection in the majority of cases and an inpatient rate of about 50% [35].

## 5. Infection Control—Low Viral Load Can Transmit Infections

In SARS-CoV-2, the relationship between viral load and transmissibility is complex and varies among different SARS-CoV-2 variants, and it is also influenced by vaccination status. Transmission is also shaped by factors such as virus variants that evade immunity, the limited immune response in older individuals, and heightened transmission risks among immunocompromised patients despite vaccination [36,37,38]. Further, the gastrointestinal route of transmission was initially undervalued during the pandemic [39]. Some epidemiological studies, but not all, show that transmission from vaccinated individuals with low viral load can occur, with the degree of transmission depending on the specific variant, vaccine type, and time since vaccination. Unvaccinated individuals infected with the Delta variant show higher infectiousness, accompanied by higher viral load, than those infected with variants that are yet of interest. Vaccination lowers viral load in those infected with the Delta variant, particularly in individuals who have received booster doses. However, the possibility of transmission remains. Yet, in fully vaccinated Delta-infected individuals, both infectiousness and viral load decrease within the first 5 days post-onset of symptoms, suggesting that there is a relationship between viral load and secondary attack rate. However, the current COVID-19 vaccines do not block the transmission of SARS-CoV-2, supporting the use of facemasks in symptomatic patients, even in vaccinated patients.

In breakthrough cases of the Omicron BA.1 variant, where the infected individual has a low viral load, the risk of transmitting the disease persists, though it is lower compared to the Delta variant. This reduced transmissibility and viral load are most noticeable in those who have received booster shots, suggesting the variable effectiveness of vaccines against different variants [40,41]. The emergence of the variant blurs the distinctions between vaccinated and unvaccinated individuals, raising questions about vaccine efficacy and emphasizing the importance of keeping these possibilities in mind, as well as their consequences on the risks of secondary cases, thereby highlighting the need for diagnostic tests tailored to these situations [42,43]. Altogether, evidence supports the clinical significance of low viral loads in influenza, RSV, and SARS-CoV-2 transmission, making highly sensitive tests mandatory.

## 6. Quality—Samples and Viral Mutations Affect Test Sensitivity

Sampling sites can significantly affect test accuracy [44], with bronchoalveolar lavage samples showing the highest detection rates (93%) for SARS-CoV-2 compared to sputum, nasal, and pharyngeal swabs [20]. In contrast, for influenza strains H1N1, H3N2, and 2009-pandemic H1N1, nasopharyngeal aspirate and nasal swabs consistently produce high detection rates, whereas throat swab samples are less likely to test positive [45]. False negatives often stem from poor timing or collection techniques, particularly with nasopharyngeal swabs, which also yield lower viral titers for RSV, potentially underestimating its prevalence [20,46]. In addition, a crucial element influencing the performance of a test, which is rarely analyzed, is the impact of the pre-analytical phase. The quality of the sample collection, transport conditions, and storage conditions significantly affect the sensitivity and sometimes even the specificity of diagnostic tools [47,48]. This underscores why laboratories focus on rigorous training and procedures, especially for sample collectors [49]. The skillset of collectors hugely influences the quality of nasopharyngeal swabs and can lead to false-negative PCR results, especially when the viral load is low [49,50]. In certain studies, variations in sample quality have led to a performance decrease of more than 20%. There is an almost linear relationship between the number of cells contained in a nasopharyngeal sample and the probability of detecting a respiratory virus such as SARS-CoV-2 [44].

Far more than DNA viruses, RNA viruses are prone to genetic mutations. SARS-CoV-2 and RSV share a comparable mutation rate (around 1.10 × 10^−3^ mutation/site/year), which is much lower than the influenza mutation rate (around 1.10 × 10^−5^ mutation/site/year) [51]. The impact of these mutations on diagnostic tests has been shown to be significant. Influenza A and B exhibit two main phenomena, antigenic drift and antigenic shift, which impact diagnostic test performance. Antigenic shift, specific to influenza A, can introduce entirely new subtypes of influenza A viruses, such as A(H1N1)pdm09, to which existing tests may have no reactivity, potentially leading to complete failure in detecting these novel strains. Antigenic drift leads to incremental genetic modifications in influenza viruses, altering the surface proteins hemagglutinin and neuraminidase. These modifications can impair the ability of antigen tests to detect and bind to viral antigens effectively, resulting in a gradual decline in test sensitivity [52,53]. This is expected, as rapid antigen tests (RATs) use lateral flow technology to detect viral antigens, such as spike or nucleocapsid proteins, which could deliver results within 15–30 min [52]. PCR tests are less susceptible to these mutations as long as their design includes more than one gene target for pathogen detection [54].

Studies reveal wide variability in RAT performance in detecting SARS-CoV-2. The detection rate for the Delta variant ranges between 20 and 40%, whereas for the Omicron variant, when the Ct value is greater than 25, the sensitivity falls to between 0 and 23%. Additionally, there could be an approximate 50% reduction in RAT sensitivity in mutations from the Gamma or Beta variants, or when viral load is low [55,56]. Evidence of the impact of RSV genome mutations on the performance of diagnostic tests is less abundant. However, cases of false negatives related to these mutations have been reported [57], as well as highly variable sensitivity in antigen tests, with a pooled sensitivity below 65% [46].

## 7. Recommendations in EU—PCR Tests over Antigen Tests

The reasoning outlined above reinforces EU guidelines in year-round testing. In most EU countries, RATs for respiratory viruses such as influenza and RSV generally do not hold significant diagnostic value in primary care settings (Table 1). This claim is supported by recent guidelines issued in France, which advocate for PCR tests over antigen tests due to their superior sensitivity and specificity in clinical diagnostics [58]. In Italy, antigen tests for RSV are not generally recommended for widespread clinical use; however, they are recognized for their utility in certain scenarios, such as helping to reduce unnecessary antibiotic prescriptions [59,60]. In Spain, the use of antigen tests is typically reserved for pediatric cases when rapid molecular testing alternatives are unavailable [61]. This approach is taken to expedite diagnosis while still accommodating the logistical constraints that are often present in medical settings dealing with children [14,58,61,62,63,64,65]. Further, several studies show that rapid influenza tests perform better in children, likely because younger ages correlate with higher viral loads, enhancing test accuracy [66].

Therefore, while RATs are popular due to their ease of use, quick results, and low cost—important considerations in low-resource settings, especially where the price differential could be significant [67]—point of care (PoC) molecular tests offer superior outcomes in terms of patient care, IPC, and cost-effectiveness [68]. Molecular tests are the reference diagnostic method for detecting influenza, RSV, and SARS-CoV-2 [69,70,71]. It is important to resist the inclination to substitute antigen tests for PCR tests. Rapid antigen diagnostic tests, while quicker, often do not meet the sensitivity and specificity levels of PCR tests [72,73,74]. RATS are good for individuals showing COVID-19 symptoms, particularly in the steady state of illness. However, these tests are less accurate for asymptomatic individuals, and negative results do not guarantee the absence of infection [75,76].

It should be noted that rapid PoC PCR tests for the concurrent detection of SARS-CoV-2, influenza, and/or RSV tests are available for near-patient settings, delivering accurate results within 40 min and significantly reducing the testing time compared to standard in-house PCR tests and sputum cultures [77]. When integrated into routine clinical practice, syndromic PCR panels, detecting even more respiratory pathogens, can achieve a real-world median turnaround time of just 2.6 h, streamlining diagnosis and treatment [78].

## 8. PCR Versus RAT—Sensitivity of Antigen Tests Might Not Be “Good Enough”

When diagnosing, clinicians face a critical question: just how sensitive must our tests be? Overall performances of RATs must be analyzed carefully to decipher the clinical impact of a lower sensitivity. An analysis by Brummer et al. of 194 studies involving 221,878 RATs shows the sensitivity for detecting SARS-CoV-2 is only 76.3%, suggesting that nearly one in four actual cases might miss detection [79]. Similarly, while PCR tests show better performance with a pooled sensitivity of 92.8% and a specificity of 97.6%, RATs, evaluated across 138 studies, demonstrate a lower sensitivity of 70.6%. This difference implies that up to 29.4% of COVID-19 cases could be overlooked when using RATs [80]. The results from a study assessing the sensitivity of a rapid antigen triple test for detecting SARS-CoV-2, RSV, and influenza in 263 children imply similar consequences. In clinical practice, the sensitivities are 88.9% for SARS-CoV-2, 79.1% for RSV, and 91.6% for influenza. This reduced sensitivity results in missing about 11.1% of SARS-CoV-2 cases, 20.9% of RSV cases, and 8.4% of influenza cases among the children tested [81]. Further research by Koul et al. supports this, showing that RATs for influenza almost always yield positive results when Ct values are below 21, illustrating a clear correlation between lower Ct values and higher test positivity rates [82]. Compared to PCR, influenza RATs were the least sensitive, detecting only about 54% of influenza A and 53% of B cases, failing to detect up to half of actual influenza cases, according to a review of 162 studies by Merckx et al. [83]. In contrast, PCR tests perform well when compared to the reference tests, demonstrating 100% concordance in clinical sample results and maintaining acceptable analytical performance within their stated limits of detection [84]. A multiplex PoC PCR panel can demonstrate a sensitivity of 97.2% for SARS-CoV-2, 95.3% for influenza A, 95.6% for influenza B, and 96.1% for RSV according to a European multicenter study [68,85].

When available in these studies, the analysis of the performance of RATs according to the Ct values of PCR is of great interest. Considering Ct values as a surrogate marker for viral loads, the samples are usually considered true positives when the Ct values are below 30, while samples that are negative show higher Ct values [79]. However, PCR tests frequently yield positive results for Ct values up to 35, aligning with French guidelines that consider a SARS-CoV-2 PCR test detecting two genes with Ct values below 37 to be a true positive [86]. Thus, while there may be considerable agreement between antigen testing and PCR at high viral loads, this agreement significantly drops at lower values. For low viral loads (Ct > 30), antigen test accuracy dramatically drops [87,88], especially in detecting acute respiratory illnesses or new SARS-CoV-2 variants. Despite variations in the values across studies, which might reflect approximations of the overall sensitivity, the trend is clear (Table 2). Sensitivities for Abbott-RAT and Roche-RAT decrease from over 95% at Ct ≤ 25 to 18.8% at Ct values of 25–30, and for Ct values ≥ 32, they are 3.0% and 6.0%, respectively. Similarly, in PoC RATs for SARS-CoV-2, lower Ct values correlate with higher accuracy and better alignment with PCR results. Sensitivity is highest at 97.9% for Ct values below 20 and 90.6% for values below 25 [89]. However, the sensitivity decreases sharply above a Ct of 25, dropping to 54.4%, and decreasing to 18.7% for Ct values above 30 (Table 2) [79].

This gap in diagnostic accuracy underscores vulnerability in managing and controlling the spread of respiratory infections, particularly in settings where IPC and prescribing specific treatments are crucial. As previously discussed, nosocomial clusters could result from failure to detect the patient’s infection upon admission [6]. Further, antigen tests might lead to a higher resource burden due to the increased need for repeat testing. Stockl et al. show that among 263,572 patients tested for SARS-CoV-2 and influenza, those who initially received antigen tests were over four times more likely to undergo additional testing on the same day compared to those who started with molecular PCR tests (70% vs. 16%) [96]. The outcome is significant costs in terms of time, personal protective equipment, and patient care expenses [97,98].

## 9. Future Perspectives in Testing

Finally, adding to the complexity, all molecular tests are not equivalent. Among the different molecular techniques—such as PCR, quantitative, real-time, multiplex, digital, or loop-mediated isothermal amplification (LAMP)—PCR and LAMP are some of the most frequently used molecular techniques. LAMP is faster in obtaining results with simplified equipment requirements, simpler, and has a lower cost; however, it is less versatile in multiplexing and has shown lower performance compared to PCR. Several studies show that the highest sensitivity of LAMP tests occurs at high viral loads. For instance, if LAMP achieved a sensitivity of 100% for Ct values below 31, this sensitivity sharply declines to 15.79% for Ct values above 36, indicating that lower viral loads severely limit the test performances [99]. Similarly, LAMP sensitivity for detecting SARS-CoV-2 RNA is insufficient for samples during the early or later stages of infection [100]. This relationship underscores the importance of viral load in determining the diagnostic accuracy of any nucleic acid amplification test and highlights the superior performance of PCR in providing accurate results under various conditions [101]. When examining the distribution of patients presenting to the emergency room with respiratory symptoms based on their viral load, the proportion of patients with low viral loads (Ct values greater than 30) is not negligible, ranging from 20% to 30% [89,95,102]. Modeling the impact of using a low-performance test compared to a high-performance test reveals striking results for the three viruses in question; over a season, among 15,000 patients tested, approximately 1200 would be incorrectly classified as negative.

## 10. Conclusions

The sensitivity of antigen and PCR tests is linked to the viral loads present in individuals, which varies due to several factors, such as the disease stage, vaccination status, and sample quality. A low viral load in infected individuals can initiate secondary cases within communities and hospitals. PCR tests can also be likewise impacted, but the influence is lower due to their comparative design and intrinsic principles. While PCR tests can accurately diagnose SARS-CoV-2, influenza, or RSV, clinicians must also account for test performance, which depends on sample quality, symptom presentation, disease prevalence, and pretest probability—factors that vary seasonally and across populations. Accurate testing performance is vital for infection control, as it enables timely treatment and isolation of cases, prevents the spread of disease, and reduces costs by minimizing unnecessary patient care, testing, and treatments. Even though the current aggressiveness of SARS-CoV-2 variants is lower, the emergence of pandemic influenza strains, RSV variants selected by new targeted drugs, or even outbreaks caused by bioterrorism-related activities should be considered potential risks to healthcare systems. This supports the need for sustained accurate diagnostic capabilities to be integrated into healthcare preparedness policies.

## Figures and Tables

**Table 1:** Hospital PCR testing is recommended in some EU countries.

Countries	COVID-19	Influenza	RSV	Reference
France	PCR	PCR	PCR	HAS 2023 [58]
Germany	PCR	PCR	PCR	DGP 2021 [62]
Spain	Unspecified	PCR	Unspecified	SEIMC 2023 [61]
The Netherlands	PCR	PCR	PCR	RIVM 2023 [64]
Belgium	PCR	PCR	PCR	Belgian Health Ministry 2023 [63]
ECDC	PCR	PCR	PCR	ECDC 2023 [14]

France: Rapid antigen tests for influenza, RSV, and/or COVID-19 do not show proven medical value for diagnosing acute respiratory infections in primary care. Spain: Influenza antigen tests in children should be used only when rapid molecular tests are unavailable and should be quality-controlled by a virology laboratory. These tests are recommended within 24–48 h of symptom onset in both primary and emergency care settings. ECDC: Hospitalized patients showing symptoms of respiratory infections should be quickly tested for viruses, primarily through molecular tests like PCR, or through point-of-care tests if available.

**Table 2:** Antigen tests are less sensitive at low viral load (high Ct values) [80,89,90,91,92,93,94,95].

Ct Values	<20	≤20	20–25	≤25	≥25	25–30	≥30	>30	≤30	30–32	30–35	≥32	>33
Sensitivity (%) SARS-CoV-2	98.0–95.9	82.7	-	85.7–96.0	62.7	18.7	41.7	36.4–38.6	77.1–90.2	0–7.14	36.5	3.0–6.0	16.7
RSV	-	-	100	-	-	50–93	-	-	-	-	-	-	10–28
H1N1pdm09	93		60	-	-	26		28	-	-	-	-	-
Seasonal H1N1	-	-	78	-	-	67	-	--	-	-	-	-	-
Seasonal H3N2	-	-	100	-	-	56		0	-	-	-	-	-

- No value.

## Data Availability

No new data were created or analyzed in this study. Data sharing is not applicable to this article.
